# Gaps between current clinical practice and evidence-based guidelines for treatment and care of older patients with Community Acquired Pneumonia: a descriptive cross-sectional study

**DOI:** 10.1186/s12879-019-4742-4

**Published:** 2020-01-23

**Authors:** Signe Eekholm, Gerd Ahlström, Jimmie Kristensson, Tove Lindhardt

**Affiliations:** 1 Department of Health Sciences, Faculty of Medicine, Lund University, P.O. Box 157, SE-221 00 Lund, Sweden; 2 Department of Health Sciences, Lund University, P.O. Box 157, SE-221 00 Lund, Sweden; 3 Research Unit for Clinical Nursing, Department of Internal Medicine, Copenhagen University Hospital Herlev and Gentofte, Herlev Ringvej 75, 2730 Herlev, Denmark

**Keywords:** Community acquired pneumonia, Evidence-based guidelines, Adherence, Diagnosis, Treatment, Nursing care

## Abstract

**Background:**

Community acquired pneumonia (CAP) remains a significant cause of morbidity and in-hospital mortality, and readmission rates are rising for older persons (> 65 years). Optimized treatment and nursing care will benefit patients and the health economy. Hence, there is a need to describe gaps between current clinical practice and recommendations in evidence-based guidelines for diagnostic procedures, medical treatment and nursing interventions for older patients with CAP.

**Methods:**

Structured observations, individual ad hoc interviews and audits of patient records were carried out in an emergency department and three medical units. Data were analysed by manifest content analysis and descriptive statistics.

**Results:**

Thirty patients (median age 74 years) admitted with CAP and 86 physicians, nurses, physiotherapists were included. The median length of stay (LOS) was 6.5 days, in-hospital mortality was10 and 40.7% were readmitted within one month. The severity assessment tool (CURB-65) was used in 16.7% of the patients, correct antibiotic treatment prescribed for 13.3% and chest radiography (≤6 weeks post-discharge) prescribed for 22.2%. Fluid therapy, nutrition support and mobilisation plans were found to be developed sporadically, and interventions to be performed unsystematically and sparingly. Positive Expiratory Pressure therapy and oral care were the nursing interventions with lowest adherence, ranging from 18.2 to 55.6%.

**Conclusions:**

Adherence to recommendations was low for several central treatment and nursing care interventions for patients with CAP with possible consequences for patients and the use of resources. Thus, there is an urgent need to identify and remove barriers to adherence to recommendations in the neglected areas in view of the potential to improve patient outcomes.

## Introduction

There is a strong consensus worldwide that Community Acquired Pneumonia (CAP) remains a significant cause of morbidity and mortality and that the incidence is highest in older persons (> 65 years) [1, 2]. In Denmark, CAP is the fifth most common cause of acute hospitalization among older patients (> 67 years) and the most common reason for readmission [3, 4]. In 2016, more than 31,500 older persons (> 65 years) were admitted to Danish hospitals with CAP and admission rates are rising at 1% per year [5] due to the overall increase in the older population [6]. Therefore, CAP represents a major cost and capacity challenge for hospitals and society.

Much effort has gone into developing national [7–11] and international [12–15] evidence-based guidelines (EBG) for diagnostic procedures, medical treatment and general management i.e. nursing care of patients with CAP, based on systematic review of scientific evidence and best practice. They are an important aid in translating scientific evidence into daily practice and have been developed to support healthcare professionals (HPs) in decision-making about appropriate and effective treatment and care for CAP patients [16]. An international cohort study [17] found that when HPs adhere to EBG for CAP, patient outcomes are significantly improved, with decreased length of hospital stay, in-hospital mortality and time to clinical stability. However, a Danish national cohort study [4] found large variations within regions and between hospitals in length of stay (LOS), in-hospital mortality, post-discharge mortality and readmission within 30 days.

This suggests an uneven and insufficient implementation of EBG. Based on these findings, a related study [18] investigated patient records (*n* = 100) of the same cohort from 20 hospitals across the country. They found that, while diagnostic procedures and medical treatment for CAP patients mainly concurred with EBG, no systematic plan for treatment or care was documented and general management recommendations for nutritional care, mobilisation and physiotherapy were neglected or only sporadically addressed. This represents a serious threat to patient safety [19, 20] indicating the need to improve the quality and effectiveness of treatment and care as well as the implemntation of evidence in practice [21, 22]. Hitherto, the adherence to EBG for treatment and care of patients with CAP in national studies has been investigated mainly by audits [4, 18]. However, as the lack of documentation is a well-described problem [18, 23], audits of patient records do not necessarily reflect the actual treatment and care. Hence, the possibility of evaluating the actual level of adherence to EBG by audit of patient records is limited [23], and observations of clinical practice are called for to identify gaps between current clinical practice and the recommendations in EBG for treatment and care of patients with CAP.

### Aim

The aim of this study was to identify gaps between current clinical practice and evidence-based recommendations regarding diagnostic procedures, medical treatment and general management (nursing care interventions) for older patients admitted with CAP.

## Materials and methods

### Design

This study had a descriptive cross-sectional design based on structured participant observations, individual ad hoc interviews during observations and audits of patient records. It is the first of four studies applying the systematic approach of implementation research [22] in which the first step is to investigate current clinical practice compared to the recommendations in EBG.

### Setting

The study was conducted in a 957-bed university hospital in the capital region of Denmark that serves an area of 700,000 inhabitants providing specialist healthcare to approximately 174,000 patients per year. Patients who arrive at the hospital with suspected CAP are examined, diagnosed and stabilized in the emergency department (ED). The ED comprises a unit for internal medicine, staffed by nurses and physicians from the department of internal medicine covering various specialties. Stabilized patients in need of further treatment and care (>24 h), are transferred to one of three units (infectious diseases, respiratory diseases, or short-term unit) in the department of internal medicine, depending on which unit has a spare bed. On arrival from ED to the medical unit, the nurses assess the patients for individual care needs utilizing validated screening tools (e.g. functional level, nutritional status, etc.) [24] in order to develop individual treatment and nursing care plan. Regional guidelines require this plan to be made within 24 h of admission [8, 9, 11]. When reduced functional ability or malnutrition is identified, nurses need to develop care plans in cooperation with physiotherapists or dieticians. Likewise, the physicians assess each patient’s response to the medical treatment and the microbiology results and modify it accordingly within 48–72 h of admission.

### Sample and recruitment

Patients (≥65 years) admitted with CAP and the staff who cared for them in the ED and the three medical units, were included prospectively and consecutively in the six months period of September 2016 to February 2017.

#### Patient group

**Fig. 1:**
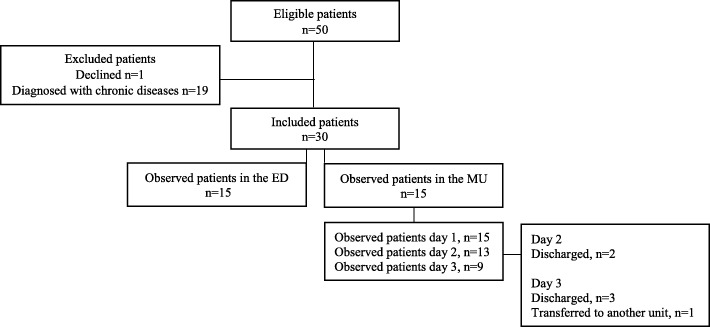
Flow charge of included patients

In total, 50 patients were invited to participate the study. The sample was recruited in two phases: firstly, from the ED and secondly from the three medical units (MU). The inclusion process was managed by assessing the patient lists and patient records for eligible patients. At the ED, patients were selected at their arrival and if they had one or more of following symptoms: confusion, cough, expectoration, dyspnoea, fever, respiratory pain, tachypnoea, or lung crepitus. Inclusion was made when patients were diagnosed with CAP and given the ICD-10 code (International Statistical Classification of Diseases and Related Health Problems - Tenth Revision) DJ09.0-DJ18.9. At the MU, patients were approached if they were ≥ 65 years and diagnosed with CAP. Patients were excluded if they were not able to give informed consent (e.g. due to severe confusion) or had been diagnosed with chronic respiratory diseases (e.g. COPD, asthma etc.). Of 50 patients, one refused, four had severe confusion and 15 were excluded due to diagnosis with other respiratory diseases than CAP. The selection of the patients consisted of 30 patients (≥65 years) admitted with CAP, *n* = 15 patients from the ED and 15 patients from the MU (Fig. 1).

#### Staff group

The HPs in the selected units received information of the project at staff meetings and by the unit management’s weekly newsletter. Once each patient was recruited the HPs who were treating and caring for them were identified and asked to participate. HPs had the right to refuse to participate in observations and interviews, but none did so. A total of 86 HPs (40 physicians, 40 nurses and 6 physiotherapists) delivered healthcare for the patients during the project period and were observed during the study.

### Data collection methods

Data were collected through structured participant observations and individual ad hoc interviews, and through review of patient records. A data collection guide was developed for this study and structured according to the EBG criteria for diagnostic procedures, medical treatment and general management for patients with CAP [7–15], as shown in Additional file 1. General management i.e. nursing care interventions focused on: sputum mobilisation, oral care, fluids, nutrition, mobilisation and oxygen therapy, [7–15] all of which are central to nursing care and delivered by nurses.

In order to be able to identify if the relevant and sufficient treatment and care had been initiated in accordance with the patient’s individual needs (i.e. adherence to EBG), patient needs were assessed independently of HP assessments by the researcher (SE), regarding the patient’s hemodynamical and clinical status and according to the criteria in the EBG. This comprised specifically: identification of CURB-65 score, travel exposure, relevance of sputum test and arterial blood gases, right choice of antibiotic treatment and all general management interventions (Additional file 1).

#### Structured participant observations and individual ad hoc interviews

*The structured participant observations* were carried out in two parts, following the data collection guide described above in order to identify gaps between current clinical practice and EBG recommendations regarding diagnostic procedures, medical treatment and general management interventions.

1. The first part took place in the ED, where the observation focus was on HPs’ adherence to the guidelines for diagnostic procedures and acute treatment and care. Observations began at each patient’s arrival to ED and ended when diagnosis was established, treatment plans made, and the medical treatment was initiated. The individual observations ranged between 6 and 8.5 h.
2. The second part was carried out in the MUs, with focus on nurses’ adherence to recommendations for general management (nutrition, fluids, mobilisation, sputum mobilisation, and oral care management). The individual observations lasted from 07:30 to 19:00–21:00 for three days or until the patient was discharged, admitted to ICU or to another non-medical unit. For these reasons 15 patients were observed for one day, 13 for two days and 9 for three days (Fig. 1). In total, 528 h of observations were carried out. Field notes were continually written by hand during the observations in chronological order and included information about the date, time and the place of the observation, the context, the participants, verbatim verbal exchanges and researcher’s personal reflections. Field notes were transcribed verbatim.

*Individual* ad hoc *interviews* with patients and HPs were conducted during observations in order to clarify any uncertainties. Notes were taken by hand during the interviews and transcribed verbatim as complementary data to the observational field notes. In total, 116 study participants (30 patients with CAP and 86 HPs) were interviewed.

#### Patient record reviews

*Patient records* were reviewed retrospectively according to a data collection guide to extract relevant variables including data for patient characteristics. This comprised: The Charlson Comorbidity Index [25] to calculate the impact of co-morbidities, the Cumulated Ambulation Score [26] assessing patients’ basic mobility on admission and the CAP severity scoring system, CURB-65 score [13, 14] assessing the risk of mortality and severity of CAP. Further variables extracted from patient journals were: diagnostics procedures, medical treatment, discharge management and general management (Additional file 1). Furthermore, data for readmission, hospital mortality and mortality within 1 month of hospitalization were collected through patient records and data were recorded into a tally sheet created in a Word file (Additional file 2).

Credibility was aimed at by involving an experienced clinician and senior researcher (last author) in reflections at several key stages during the data collection period, as well as in all phases of the data analysis, challenging the first author’s preunderstanding, choices and interpretations. The two additional authors participated in discussions and evaluation of the process at several stages of the data collection to decide whether any adjustments or changes were needed.

### Data analysis

The transcribed field notes, interview texts and patient record reviews were analysed as one text body. Data were analysed by deductive, quantitative manifest content analysis [27]. The text was read through and coded according to analytic categories consisting of the criteria from the EBG. Each coding was given the value 0 or 1; 0 being non-compliant and 1 being compliant with the respective criterion in the EBG. The SPSS version 25 software was used for descriptive statistics analysis.

To achieve a consistent approach, the first three transcribed texts were reviewed independently by all authors (SE, GA, JK, and TL). The deductive manifest analysis process was discussed until the authors reached a common understanding and agreement of the further analysis process. Both the first and last authors read all transcribed texts independently, wrote memos with reflections that were discussed and compared to reach a common understanding of the content. The first author (SE) independently established the manifest content and carried out the descriptive statistics, which was assessed and approved by the last author (TL).

The text was read through and coded according to analytic categories consisting of the criteria from the EBG. Each coding was given the value 0 or 1; 0 being non-compliant and 1 being compliant with the respective criterion in the EBG. The SPSS version 25 software was applied to record the data for the use of descriptive statistics.

## Results

### Patient characteristics

The characteristics of the 30 patients included in the study are shown in Table 1. The median age for the patients was 74 years and 16 (53.3%) were males. The median level of co-morbidities was two. The most common findings at the admission were increased C-reactive protein in 28 patients (93.3%), dyspnoea in 26 (86.7%), cough in 25 (83.3%) and sputum production in 25 (83.3%). Nutritional deficiencies were presented in 23 (76.7%) patients and 10 (33.3%) were dependent on personal assistance because of decreased functional level. The CURB-65 score indicated that 13 (43.3%) patients had moderate severity of CAP and 8 (26.7%) had severe CAP and high risk of mortality. Two patients were discharged within 24 h of admission. The median length of hospitalization was 6.5 days and three (10%) patients died while admitted. Of 27 surviving patients, 11 (40.7%) were readmitted within one month. The incidence of 30 days mortality was 7.4%.

**Table 1:** Characteristics of patients with CAP at admission to hospital (*n* = 30)

Diagnostic characteristics	Frequency, n (%) or *median (IQR)*
Gender/male,	16 (53.3)
Age, *median (IQR)*	*74 (69.8–84.5)*
Smokers	5 (16.7)
Penicillin allergy	1 (3.3)
Cumulated Ambulation Score (CAS) < 6	10 (33.3)
Charlson Comorbidity Index (CCI), *median (IQR)*	*2 (1.0–3.0)*
Nutritional deficiencies,	23 (76.7)
Fever (≥38 °C)	13 (43.3)
C-reactive protein (> 10 mg/L)	28 (93.3)
Sputum production	25 (83.3)
Dyspnoea	26 (86.7)
Chills	4 (13.3)
Pleuritic chest pain	3 (10.0)
Confusion	7 (23.3)
Pulse rate > 100	13 (43.3)
Respiratory rate ≥ 30 breaths/min	4 (13.3)
Cough	25 (83.3)
CURB-65 score: 0–1 points	9 (30.0)
CURB-65 score: 2 points	13 (43.3)
CURB-65 score: ≥3 points	8 (26.7)
Recent travel (≤2 weeks)	3 (10.0)
Responding to treatment ≤48 h	20 (71.4)^a^
Length of hospital stay, *median (IQR)*	*6.5 (3.0–14.3)*
In-hospital mortality	3 (10.0)
Readmission ≤1 month	11 (40.7)^b^
Post discharge mortality ≤1 month	2 (7.4)^b^

^a^Adjusted for 2 patients who were discharged ≤24 h of admission

^b^Adjusted for in-hospital mortality on 27 patients

CCI = Charlson Comorbidity Index is calculated based on 19 predefined comorbidities with assigned weights of 1, 2, 3, or 6 and summed to the total CCI score. CAS=Cumulated Ambulation Score is calculated by assessing 3 activities (1. getting in and out of bed, 2. sitting down and standing up from a chair, 3. walking ability with or without a walking aid) on a three-point scale

(2 = independent of human assistance or guidance, 1 = requiring human assistance or guidance to perform activity, 0 = unable to perform activity despite human assistance). The score for each activity is cumulated to provide a daily range from 0 to 6. CAS score < 6 indicates dependent of human assistance. CURB-65 = severity assessment score is calculated by giving 1 point for each of the following prognostic features: Confusion, Urea>7 mmol/l, Respiratory rate >30 breaths/minute, Low systolic (<90 mmHg) or diastolic (<60 mmHg) blood pressure, ≥65 years. The total sum is stratified for CAP severity: (score 0–1) low severity; (score 2) moderate severity; (score 3–5) high severity

### HP’s adherence to diagnostic procedures regarding EBG criteria

All 30 patients received: chest examinations to detect rales or bronchial breath sounds, oxygen saturation, electrolytes, C- reactive protein, full blood count, liver function test and mini-mental test (Table 2). Chest radiography was carried out for 28 (93.3%) patients and blood cultures for 26 (86.7%) patients. Adherence to the recommended procedure was achieved regarding arterial blood gases in 24 of 26 (92.3%) patients, sputum culture in 19 of 25 (76.0%) and sensitivity test in 7 of 13 (53.8%) patients respectively. The CURB-65 was applied in 5 (16.7%) of the 30 patients. After 48 h, two patients were stabilized and discharged. Of the 28 admitted patients, 8 (28.6%) did not respond to the treatment within 48 h. In repeated diagnostic procedures for non-responding patients’ adherence was highest for C-reactive protein test, white cell count, chest radiography and blood cultures. Adherence was lowest for Polymerase Chain Reaction test (PCR), sputum test for culture and sensitivity, *Legionella pneumophila* urine antigen test and Pneumococcal urine antigen test.

**Table 2:** HPs’ adherence to diagnostic tests regarding EBG criteria for patients with CAP

Diagnostic tests and tests for non-responding patients	Patients n	Exposed for test, n (%)
Diagnostic tests:		
Chest radiography	30	28 (93.3)
Sputum test for culture and sensitivity	25	19 (76.0)^a^
Chest examination (auscultation/percussion)	30	30 (100.0)
Oxygen saturation	30	30 (100.0)
Mini mental test	30	30 (100.0)
Travel exposure anamnesis	13	7 (53.8)^b^
Blood tests:		
Blood cultures	30	26 (86.7)
Full blood count	30	30 (100.0)
C-reactive protein	30	30 (100.0)
Electrolytes	30	30 (100.0)
Liver function	30	30 (100.0)
Arterial blood gases	26	24 (92.3)^c^
Appliance of CURB-65 score to assess disease severity	30	5 (16.7)
Non-responding to treatment ≤48 h	28	8 (28.6)^d^
Tests for non-responding patients:		
L. pneumophila urine antigen test, LUT	8	1 (12.5)
Pneumococcal urine antigen test, PUT	8	1 (12.5)
Polymerase chain reaction test, PCR	8	3 (37.5)
Sputum test for culture and sensitivity	8	1 (12.5)
C-reactive protein and white cell count	8	8 (100.0)
Chest radiography	8	7 (87.5)
Blood cultures	8	7 (87.5)

^a^Adjusted for patients with sputum

^b^Adjusted for patients not able to travel

^c^Adjusted for patients with SpO_2_ < 92%, RF > 20 or dyspnoea

^d^Adjusted for patients who were discharged ≤24 h of admission

### HP’s adherence to medical treatment and discharge regarding EBG criteria

The median time for intravenous antibiotic prescription was 165.5 min and 235.5 min to initiation of medication administration (Table 3). Antibiotics (AB) were prescribed in accordance with EBG recommendations for four (13.3%) patients. Adjustment of AB treatment in accordance to pathogen resistance test (≤48 h) was reached in 22 (78.6%) cases. The expected response to treatment within 48 h occurred in 20 (71.4%) cases (Table 1), and the recommended switch from intravenous antibiotic treatment to oral treatment within 48–72 h for responding patients was carried out in 15 (75.0%) cases (Table 3). The six weeks clinical control was prescribed in 21 (77.8%) cases. Control chest radiography was required in all cases but prescribed in six (22.2%).

**Table 3:** HPs’ adherence to antibiotic treatment and discharge management regarding EBG criteria

Antibiotic treatment and discharge management	Patients n	Adherence
Antibiotic (AB) treatment		
Time (minutes) to AB prescription, *median (IQR)*	30	*165.5 (127.3–255.0)*
Time (minutes) to AB administration, *median (IQR)*	30	*235.5 (187.0–401.0)*
AB treatment prescribed in accordance with EBG criteria, n (%)	30	4 (13.3)
Adjustment of AB treatment ≤48 h of admission, n (%)	28	22 (78.6)^a^
Switch from intravenous to oral AB treatment in accordance with EBG criteria, n (%)	20	15 (75.0)^b^
Discharge management		
Prescription of 6 weeks polyclinical control, n (%)	27	21 (77.8)^c^
Prescription of chest radiography (≤6 weeks post-discharge), n (%)	27	6 (22.2)^c^

^a^Adjusted for patients who were discharged ≤24 h of admission

^b^Adjusted for patients who responded to treatment ≤48 h (*n* = 20)

^c^Adjusted for in-hospital mortality (*n* = 27 surviving patients)

### HP’s adherence to general management regarding EBG criteria

Of the 11 patients who fulfilled the EBG for sputum mobilization support, two (18.2%) patients received it by Positive Expiratory Pressure (PEP) on the first observation day and three (42.9%) patients on the third observation day (Table 4). Oral care was delivered twice a day to four (26.7%) patients on the first day of observation, three (23.1%) on the second and five (55.6%) on the third observation day. Of ten patients who needed fluid therapy, the fluid therapy plan was developed for nine (90.0%) patients on the first observation day, eight (88.9%) on the second day, and six (85.7%) on the third observation day. Of those patients, four received fluid therapy according to their plan during the first and last observation days and two (25.0%) on the second observation day. Of the total sample, eight patients were screened for nutritional status, four of them within 24 h of admission. Eleven (73.3%) patients fulfilled the EBG criteria for nutritional support at the first observation, nine (69.2%) at the second and seven (77.8%) on the third day. In total, a nutritional support plan was developed for six patients. Due to lack of documentation of energy and protein needs for all patients included from MU, and insufficient registration of nutritional intake in the patient journals it was not possible to assess whether patients received nutrition in accordance with their needs. Mobilisation ≥20 min was achieved for 11 (73.3%) patients during the first observation day, eight (61.5%) during the second day and five (55.6%) during the third. For patients needing mobilisation support, a mobilisation plan was developed for six (60.0%) patients at the first and the last observation day, and for seven (75.0%) patients during the second day. Two (33.3%) patients were mobilised according to their individual mobilisation plan on the first observation day, three (42.9%) on the second day and three (50.0%) on the third. Oxygen therapy was delivered according to recommended criteria in EBG for eight (88.9%) patients during the first observation day and 100.0% during subsequent observation days.

**Table 4:** HPs’ adherence to EBG recommendations for general management in three medical units*

General management	Observation day 1, Patients in need / Patients receiving treatment, n/n (%)	Observation day 2, Patients in need / Patients receiving treatment, n/n (%)	Observation day 3, Patients in need / Patients receiving treatment, n/n (%)
Sputum mobilisation			
In need of sputum mobilisation	15/11 (73.3)	13/10 (76.9)	9/7 (77.8)
Sputum mobilisation received by PEP^**b**^	11/2 (18.2)	10/2 (20.0)	7/3 (42.9)
Oral care			
Oral care (twice a day)	15/4 (26.7)	13/3 (23.1)	9/5 (55.6)
Intravenous fluid therapy			
In need of fluid therapy	15/10 (66.7)	13/9 (69.2)	9/7 (77.8)
Fluid therapy plan developed^**c**^	10/9 (90.0)	9/8 (88.9)	7/6 (85.7)
Fluids received accordance to fluid therapy plan^**c**^	9/4 (44.4)	8/2 (25.0)	6/4 (66.7)
Nutrition support			
Screening for nutrition status (≤24 h)	15/4 (26.7)	13/4(30.8)	9/4 (44.4)
Screening for nutrition status (up to or during observation days)	15/ 8 (53.3)	13/8 (61.5)	9/8 (88.9)
In need of nutrition support	15/11 (73.3)	13/9 (69.2)	9/7 (77.8)
Development of nutrition support plan^d, b^	11/6 (54.5)	9/6 (66.7)	7/6 (85.7)
Mobilization			
Mobilization (walk or sit out of bed ≥20 min)	15/11 (73.3)	13/8 (61.5)	9/5 (55.6)
In need of mobilisation support	15/10 (66.7)	13/10 (76.9)	9/8 (88.9)
Development of mobilisation plan^e, b^	10/6 (60.0)	10/7 (70.0)	8/6 (75.0)
Mobilization received accordance to mobilisation plan^e^	6/2 (33.3)	7/3 (42.9)	6/3(50.0)
Oxygen therapy			
In need of oxygen therapy	15/9 (60.0)	13/9 (69.2)	9/8 (88.9)
Oxygen therapy initiated and monitored according to SpO_2_ level^a^	9/8 (88.9)	9/9 (100.0)	8/8 (100.0)

^a^Overall, 15 patients were observed day 1, 13 patients day 2 and 9 patients day 3. Results are expressed as a number of patients expected to receive the intervention and the number of patients who received it

^a-e^Test is adjusted for patients in need of: a) oxygen therapy; b) sputum mobilisation; c) intravenous fluid therapy; d) nutrition support; e) mobilisation plan

^b^Care plan was not developed according to EBG but was accepted because it described some sort of plan for patient, although incomplete and unsystematic

## Discussion

The aim of this study was to identify gaps between current clinical practice and EBG recommendations regarding diagnostic procedures, medical treatment and general management for older patients with CAP. We identified a few, but potentially serious gaps in diagnostic procedures and medical treatment, as well as in general management, all of them with possible impact on patient outcomes and safety.

### Diagnostic procedures and medical treatment

Among diagnostic procedure recommendations, the severity assessment score CURB-65 was rarely used within the units included in this study. This result is consistent with previous national and international research findings [18, 28–30]. Initial assessment of illness severity is considered to be one of the most important steps in the management of older patients admitted with CAP [7, 11–14]. It supports HPs in determining patient needs at admission, site of care, the extent of diagnostic testing and in choosing the appropriate antibiotic treatment, all of which are independent factors with impact on CAP patient morbidity and mortality [7, 11–14]. Our results and previous studies indicate that CURB-65 is not routine practice in hospital settings and tailored implementation strategies are needed [22] to achieve higher adherence rates for the benefit of patient safety and the health economy.

Among medical treatment procedures, the initial choice of antibiotics was the intervention that was least frequently carried out according to recommendations. Presumably, this is a consequence of the low prevalence of severity assessment, as the initial choice of antibiotic treatment should be guided by the CURB-65 score [7, 11–14]. Appropriate antibiotic prescription in hospitals ensures effective treatment of patients and administration of appropriate antibiotics within 4–8 h is associated with 5–43% relative reduction in mortality [31, 32]. Inappropriate antibiotic therapy, on the other hand, is an independent predictor of in-hospital and 30-day mortality and associated with morbidity and increased treatment costs [31, 33–35]. Regrettably, inappropriate antibiotic treatment is common, both when it concerns the initial choice of AB [36, 37] and treatment targeted the identified pathogen [33]. A Cochrane review [38] finds strong evidence for the effect on compliance of interventions that support physicians in prescribing the appropriate treatment (e.g. procedural instructions, feedback and stewardship). Further effects were reduced duration of antibiotic treatment by 1.95 days (95% CI 2.22 to 1.67) and length of stay by 1.12 days (95% CI 0.70 to 1.54).

Timely switch from intravenous to oral antibiotic treatment was seen in most cases in our study. Early switch of antibiotic treatment not later than the third day may reduce the iatrogenic events and LOS by 3.4 days [39, 40]. Importantly, a quarter of our sample were not switched to oral treatment in time despite clinical and haemodynamical stability as recommended in EBG [7, 11–14], with possible consequences for patient safety and economy [39]. Therefore, there is also a need to identify barriers to early switching and to develop a tailored strategy for the implementation of an intervention that facilitates timely switching to oral treatment.

More than a quarter of the patients in our study did not respond to treatment within the expected time frame, and a systematic diagnostic approach for non-responding patients was limited. It is common to find patients not responding to treatment, and mortality rates for those patients are reported as high as 49% [13]. Therefore, it is strongly recommended to reassess non-responding patients’ treatment and to perform multiple relevant diagnostic tests to determine specific respiratory pathogens, in order to permit prescription of appropriate antibiotic treatment [7, 11–14]. According to Arancibia et al. [41] a systematic diagnostic approach by invasive, non-invasive, and imaging procedures can lead to a specific diagnosis in 73% of cases. Hence, a systematic and evidence-based approach to avoid treatment failure and reduce the rate of in-hospital mortality seems warranted.

### General management

General management i.e. nursing care interventions, performed by nurses in our study, are vital for patients with CAP as they are reported to have reduced morbidity, mortality, LOS and readmission rates [15, 42–51].

The only nursing care intervention that was performed systematically in our study was oxygen therapy, whereas sputum mobilisation by PEP and oral care were carried out less frequently. Previous studies have identified that PEP treatment can reduce fever duration and length of hospital stay [42, 48] and oral care in critically ill patients is associated with 18 to 24% of reduction in the odds of developing ventilator-associated pneumonia [51], while low adherence to oral care has consequences such as pain, malnutrition, readmissions, increased healthcare costs and mortality [10, 46, 51]. Therefore, special attention should be paid to sputum mobilisation and oral care to achieve better adherence among HPs, in order to deliver effective and safe treatment for patients with CAP.

Despite plans for fluid therapy made for most patients in our study, adherence to planned interventions were rare and unsystematic, which can put the patient at risk of renal and electrolyte complications. According to Guppy et al. [52] the incidence of hyponatremia is common for patients with infections of the lower respiratory tract and 10.5% of CAP patients have been identified as developing hyponatremia during hospitalization. Those results indicate the need to focus on fluid therapy when designing a strategy to implement evidence-based practice for patients with CAP.

The majority of the patients in our study were at nutritional risk but, as nutrition support plans were either lacking, or at best insufficient (e.g. missing calculation of individual needs for energy and protein for all 30 patients), the nurses could not assess the sufficiency of the patients’ intake nor could the researchers assess HPs’ adherence to recommendations for nutrition support. Therefore, adherence to nutrition support needs to be determined with further research.

In our study, more than half of the patients were mobilised 20 min or more. Noting that EBG strongly recommend 20 min mobilisation within 24 h of hospitalisation and increase of mobilisation each subsequent day, our results indicate that approximately a quarter of the patients were mobilised less than recommended. Even though mobilisation plans were frequently developed, activities according to mobilisation plans were less frequently performed. Those results are consistent with other studies reporting that older patients with CAP may be at risk of functional loss during hospitalisation and after discharge, due to insufficient mobilisation [20, 53]. Considering that regular mobilisation reduces functional decline, mobilisation should be encouraged for older patients with CAP during hospitalisation [54]. Moreover, studies of CAP demonstrate that early mobilisation is safe and effective in reducing length of hospital stay [45, 55].

To deliver effective and targeted nursing care interventions, a systematic assessment of patient individual needs and development of an individual nursing care plan is essential [7–15]. In our study, nursing care plans were found to be scarce and unsystematically developed (e.g. missing data on patient status, developed only partly, intervention or duration of the intervention not described according to EBG recommendations). This supported previous finding by Lindhardt et al. [18] who found care planning for patients with CAP to be rare and unsystematic and nursing documentation insufficient. Jones et al. [47] identified also nursing care planning to be among the top five most frequently incomplete activities in nursing practice. On the other hand, an observational study by De Marinis et al. [23] has reported that only 40% of nursing activities are consistent with the documentation as nurses perform more activities than they report. This result is supported by findings from the recent systematic review [56] that found nursing care planning to be more often missed than performance of nursing care. The level of missed nursing care associated with adverse patient outcomes is high, with an overall estimate of 88% in acute hospitals in Europe [56]. The association between nursing care planning and the level of interventions performed was not under investigation in our study but deserves more specific attention in future studies.

The lack of systematic care planning and management of described interventions constitutes a threat to patient safety. Nevertheless, our findings are not breaking news as the phenomenon of *missing care,* defined as any aspects of care that is omitted or delayed, in part or in whole [56], is a comprehensive problem nationally and internationally with a prevalence of 55–98% in acute care hospitals [47, 49, 50]. Seemingly, among all the EBG recommendations in our study, nurses had more difficulties adhering to EBG than the physicians. Due to the scope of the study, the cause of the low adherence is unknown and needs further investigation in future studies. Other studies have identified staffing, time scarcity, resources, inadequate support from peers, professional behaviour, knowledge and culture as some of the factors that could influence HPs’ adherence to EBG [34, 56–58]. Further, the insufficient and inadequate description of nursing interventions in the EBG for treatment and care of CAP could also be considered a barrier to nurses’ adherence to EBG recommendations. While national and international EBG for CAP thoroughly review diagnostic tools and choice of antibiotic treatment, they do not emphasise the importance of nursing care interventions and the consequences of missed care. Considering the impact of nursing interventions for patient recovery and safety [59, 60], the absence of description of nursing interventions in EBG constitutes a threat to successful patient outcome. Therefore, the revision of the EBG should be considered. However, our results indicate that even if diagnostic procedures and medical treatment are well described in the EBG, only a fraction of these guidelines have been implemented in clinical practice. This could indicate that even a thorough description of nursing interventions in the EBG may not increase the adherence rate. Instead, according to implementation researchers [21, 22] factors influencing HPs adherence to EBG should be focused on in order to promote the successful uptake of research findings into routine practice and improve the quality and effectiveness of treatment and care. Implementation science emphasizes that factors influencing HPs’ adherence to EBG can be linked to both individual, team and organizational level and are related to the context where the treatment and care is performed [22, 61]. The context is recognized as a core factor that influences implementation [22, 62]; thus, it is important to identify contextual barriers hindering, and facilitators supporting HPs in performing evidence-base practice. This is considered a fundamental criterion for successful implementation as this knowledge is crucial for the development of tailored and context-oriented implementation strategies targeting the problem areas [21, 22, 63].

#### Methodological considerations

The strength of this study was the triangulation of research methods [64] where observations, interviews and data from patient records allowed us to reveal different perspectives of a research question and helped us to achieve better understanding of the real-life management of older patients with CAP in a hospital setting. Particularly, individual ad hoc interviews were helpful to complete the data collection, as interviews revealed uncertainties that were not possible to clarify by observations or by audits, e.g. clarification of whether assessment of CURB-65 had taken place, or whether oral care had been carried out. We also acknowledge the limitation that all data were collected by a single researcher. Observations were carried out only by the first author as the presence of several observers could have affected the natural context for HPs and intimidated both them and the patients. The first author is a registered nurse with many years of professional experience, including the care of CAP patients and had a pre-understanding of the context that was required to analyse and collect data in a complex setting by multiple methods. However, this inside perspective and preunderstanding can be perceived both as a methodological strength and limitation. To enhance credibility, all authors were involved in reflections and discussions throughout the data collection period, analysis and evaluation of the project to challenge the first author’s preunderstanding, choices and interpretations.

To identify HPs’ adherence to EBG recommendations, the researcher had to make her own assessment of each patient’s condition and needs. The conclusion reached in the assessment of the patient may therefore differ from that of the HPs. However, researcher’s assessment of patient conditions and needs was guided by the EBG and as such are expected to be followed by the HPs. To increase the credibility, the last author rechecked the first author’s assessment of patient individual needs according to EBG criteria and patient’s clinical data. Furthermore, after transformation of qualitative data to quantitative data, all numerical data were rechecked according to the content in transcribed text. After entering data into SPSS, all data were rechecked three times before performance of any statistics. It is also worth noting that the study was carried out in a single hospital and the sample size for participating patients was small; hence, the transferability of the findings may be limited as the study may be context-specific.

## Conclusion

Our results indicate that HPs adherence for recommended EBG criteria for treatment and care of patients with CAP was low for several central interventions. To improve patient outcomes, and to ensure that patients receive evidence-based treatment and care, there is a need to focus on severity assessment score, correct antibiotic treatment, diagnostic procedures for non-responding patients and, particularly, on nursing care interventions. Considering described gaps, future research needs to identify barriers to EBG criteria and take them into account when implementing evidence-based clinical practice in a hospital setting.

## Supplementary information

**Additional file 1.** Recommended interventions for older patients admitted with CAP

**Additional file 2.** Tally sheet for registration

## Data Availability

All data generated and analysed during this study are included in this published article.

## Abbreviations

AB: Antibiotic
BMI: Body mass index
CAP: Community acquired pneumonia
CAS: Cumulated Ambulation Score
CCI: Charlson Comorbidity Index
CRP: C-reactive protein
CURB-65: Severity assessment score
EBG: Evidence-based guidelines
ED: Emergency department
HPs: Healthcare professionals
ICD-10: International Statistical Classification of Diseases and Related Health Problems - Tenth Revision
LOS: Length of stay
LUT: Pneumococcal urine antigen test
MU: Medical units
PCR: Polymerase chain reaction test
