# Supplementary material for: Gaps between current clinical practice and evidence-based guidelines for treatment and care of older patients with Community Acquired Pneumonia: a descriptive cross-sectional study
Source: BMC Infect Dis. 2020 Jan 23;20:73. doi: 10.1186/s12879-019-4742-4 (PMC6979078; doi:10.1186/s12879-019-4742-4)
Supplement: Supplementary file 1 — Additional file 1. Recommended interventions for older patients admitted with CAP [file 12879_2019_4742_MOESM1_ESM.docx]

**Additional file 1.** Recommended interventions for older patients admitted with CAP

| Indicator | Recommended intervention | Recommending EBG |
| --- | --- | --- |
| **Diagnostic procedures** | Chest radiography, Sputum culture and sensitivity, Physical examination of chest (auscultation/ percussion), Oxygenation saturation (SpO_2_), Mini mental test, Travel exposure (within 2 weeks).  Blood tests: Blood cultures, Full blood count, C-reactive protein (CRP), Electrolytes, Liver function, Arterial blood gases (indicated when SpO_2_ <92%, Respiratory rate >20 or dyspnoea).  Severity assessment by CURB-65 score.  Moderate or high severity score or not responding to treatment ≤48-72 hours: Chest radiography, CRP and white cell count, blood cultures, Legionella pneumophila urine antigen test (LUT), Pneumococcal urine antigen test (PUT), Polymerase chain reaction test (PCR), sputum test for culture and sensitivity. | [7, 11-14] |
| **Antibiotic (AB) treatment** | Administer AB treatment ≤4h after admission and prescribe treatment according to CURB-65 score:   1. **CURB-65 (0-2, low severity):** Benzylpenicillin 1 mill.IE (̴ 667mg) x4. Penicillin allergy: Clarithromycin 500mgx2. 2. **CURB-65 (3-5, moderate severity):** Benzylpenicillin 2 mill.IE (̴ 1333mg) x4+ and Clarithromycin 500mgx2. Penicillin allergy: Cefuroxim 500mgx3+ Clarithromycin 500mgx2. 3. **CURB-65 (≥3, high severity):** Piperacillin/tazobactam 4,0/0,5x4 + Clarithromycin 500mgx2. Penicillin allergy: Cefuroxim 1500mgx3+ Clarithromycin 500mgx2.   Adjust AB treatment accordance to pathogen resistance test ≤48-72 hours. Switch intravenous to oral treatment when: hemodynamical and clinical stability are reached, normal gastrointestinal tract for 24h and able to ingest medication. | [7, 11-14] |
| **Discharge management** | Clinical control around 6 weeks and chest radiography at around six weeks for patients ˃50 years, smokers and when symptoms persist. | [11, 12] |
| **General management:**  **Sputum mobilisation** | Airway clearance techniques by Positive Expiratory Pressure when patients have difficulty with expectoration or in the event of a pre-existing lung condition. | [11, 12] |
| **Oral care** | Tooth brushing minimum twice a day (minimum 2 minutes) with toothpaste containing 1000-1500ppm fluoride. | [10] |
| Fluid therapy | Assess volume depletion and fluid status daily. Develop target fluid therapy plan when discrepancy between fluids intake and output, electrolyte disturbances, abnormal pulse and blood pressure and in presence of: confusion, diarrhoea, vomiting. Advise to drink plenty of fluids. | [8, 9, 11, 12] |
| **Nutritional support** | Screen nutrition status within ≤24 hours of admission and develop targeted nutrition support plan for patients at risk of malnutrition (assessed by: BMI (˂20,5), weight loss ≤3 months, reduced dietary intake ≤1week, age (˃70 years) and health condition (chronic illness, bedridden, in intensive care). | [8, 9, 11, 12] |
| Mobilisation | Assess functional ability and develop targeted mobilisation plan for patients with loss of functional ability (in conjunction with hospitalisation), who need mobilisation support (for activities of daily living) or rehabilitation. Mobilize patients (walk or sit out of bed) within ≤24 hours of hospitalisation, for 20 minutes, and increase mobilisation each subsequent day. | [11, 12, 14] |
| Oxygen therapy | Oxygen therapy must be guided by the level of arterial oxygen tension (PaO_2_) and oxygen saturation (SpO_2_). For acutely ill patients (not at risk of hypercapnic respiratory failure) PaO_2_ is >8 kPa and SpO_2_ 94–98%. For patients with COPD or who are at risk of hypercapnic respiratory failure, SpO_2_ is 88-92%. | [11, 12, 15] |
