# Supplementary material for: Gaps between current clinical practice and evidence-based guidelines for treatment and care of older patients with Community Acquired Pneumonia: a descriptive cross-sectional study
Source: BMC Infect Dis. 2020 Jan 23;20:73. doi: 10.1186/s12879-019-4742-4 (PMC6979078; doi:10.1186/s12879-019-4742-4)
Supplement: Supplementary file 2 — Additional file 2. Tally sheet for registration [file 12879_2019_4742_MOESM2_ESM.docx]

# Tally sheet for registration of background data

Answer examples: yes=1, no=0, missing data=99, irrelevant=999. Data is registered by numbers in a tally sheet (in a blue box).

| **Item** | **Time** | **Date** | **Result** |
| --- | --- | --- | --- |
| Admission: time, data, unit |  |  |  |
| Transferred: time, date, unit |  |  |  |
| Discharged: time, date, unit |  |  |  |
| LOS |  |  |  |
| Gender |  |  |  |
| Age |  |  |  |
| BMI |  |  |  |
| Smoker |  |  |  |
| Penicillin allergy |  |  |  |
| CAS score |  |  |  |
| CCI score |  |  |  |
| CURB-65 score (assessed by authors) |  |  |  |
| Recent travel (≤2 weeks) |  |  |  |
| Nutrition status |  |  |  |
| In-hospital mortality |  |  |  |
| Readmission ≤1 month |  |  |  |
| Mortality ≤1 month |  |  |  |

# Tally sheet for registration of diagnostic procedures and medical treatment data

| **Item** | **Result**  **Yes=1, No=0** | **Test result** | **Time** | **Time from admission** |
| --- | --- | --- | --- | --- |
| **DIACNOSTIC PROCEDURES** |  |  |  |  |
| Symptoms at admission |  |  |  |  |
| Fever |  |  |  |  |
| Sputum production |  |  |  |  |
| Leukocytes |  |  |  |  |
| Dyspnoea |  |  |  |  |
| Chills |  |  |  |  |
| Pleuritic chest pain |  |  |  |  |
| Confusion |  |  |  |  |
| Puls rate |  |  |  |  |
| Respiratory rate |  |  |  |  |
| Cough |  |  |  |  |
| Other symptoms |  |  |  |  |
| Respiratory rate |  |  |  |  |
| Blood pressure |  |  |  |  |
| Pulse |  |  |  |  |
| Temperature |  |  |  |  |
| Oxygen saturation |  |  |  |  |
| Arterial oxygen tension |  |  |  |  |
| Total EWS score |  |  |  |  |
| Diagnostic tests performed at admission. Time |  |  |  |  |
| Chest radiography |  |  |  |  |
| Sputum test for culture and sensitivity |  |  |  |  |
| Chest examination (auscultation/percussion) |  |  |  |  |
| Mini metal test |  |  |  |  |
| Travel exposure anamnesis |  |  |  |  |
| Blood tests |  |  |  |  |
| Blood cultures |  |  |  |  |
| Full blood count |  |  |  |  |
| C-reactive protein |  |  |  |  |
| Electrolytes |  |  |  |  |
| Liver function |  |  |  |  |
| Arterial blood gases |  |  |  |  |
| Appliance of CURB-65 score to assess disease severity |  |  |  |  |
| Test for non-responding patients |  |  |  |  |
| LUT |  |  |  |  |
| PUT |  |  |  |  |
| PCR |  |  |  |  |
| Sputum test for culture and sensitivity |  |  |  |  |
| C-reactive protein and white cell count |  |  |  |  |
| Chest radiography |  |  |  |  |
| Blood cultures |  |  |  |  |
| **MEDICAL TREATMENT** |  |  |  |  |
| Time (minutes) to AB prescription |  |  |  |  |
| Time (minutes) to AB administration |  |  |  |  |
| Medication (AB) prescribed |  |  |  |  |
| Is AB prescribed correctly according to EBG |  |  |  |  |
| Is patient responding to treatment within 48 hours |  |  |  |  |
| Adjustment of AB treatment ≤48 hours of admission |  |  |  |  |
| Which AB is prescribed after adjustment |  |  |  |  |
| Is switch from intravenous to oral AB treatment in accordance with EBG criteria. |  |  |  |  |
| Reason for switch from intravenous to oral AB treatment in accordance with EBG criteria. |  |  |  |  |
| **DISCHARGE** |  |  |  |  |
| Prescription of 6 weeks polyclinical control |  |  |  |  |
| Prescription of control chest radiography ≤6weeks |  |  |  |  |

# Tally sheet for registration of general management data

| **Sputum mobilisation** | **Day 1** | **Day 2** | **Day 3** |
| --- | --- | --- | --- |
| Is patient in need for sputum mobilisation? |  |  |  |
| Has patient received sputum mobilisation by PEP, by correct technique? |  |  |  |

| **Oral care** |  | **Day 1** | **Day 2** | **Day 3** |
| --- | --- | --- | --- | --- |
| Has patient received oral care during the daytime? |  |  |  |  |
| Is performance of oral care registered in patient journal? |  |  |  |  |
| Has patient received oral care during the evening? |  |  |  |  |
| Is performance of oral care registered in patient journal? |  |  |  |  |

| **Fluid therapy** |  | **Day 1** | **Day 2** | **Day 3** |
| --- | --- | --- | --- | --- |
| Patient individual need for fluids (ml)? |  |  |  |  |
| Is patient in need for fluid therapy? |  |  |  |  |
| Is fluid therapy plan developed? What is the plan (ml)? |  |  |  |  |
| Blood tests results (electrolytes): |  |  |  |  |
| Potassium (K^+^) |  |  |  |  |
| Sodium (Na^+^) |  |  |  |  |
| Albumin |  |  |  |  |
| Creatinine |  |  |  |  |
| Carbamide |  |  |  |  |
| Fluids received (ml) |  |  |  |  |
| Intravenous fluids |  |  |  |  |
| Oral intake |  |  |  |  |
| In total |  |  |  |  |
| Has patient received fluids according to individual fluid therapy plan? |  |  |  |  |

| **Nutrition support** | **Date** | **Day 1** | **Day 2** | **Day 3** |
| --- | --- | --- | --- | --- |
| Is patient screened for nutrition status? Date |  |  |  |  |
| Is patient screened for nutrition status (≤24h at admission) |  |  |  |  |
| Is patient in need for nutrition support? |  |  |  |  |
| Is nutrition support plan developed (up to or during observation days? What is the plan? |  |  |  |  |
| Protein and kilojoule (kj) served at 7:30-11:30 |  |  |  |  |
| Protein and kj served at 11:30-17:00 |  |  |  |  |
| Protein and kj served at 17:00-24:00 |  |  |  |  |
| Intake of protein and kj at 7:30-11:30 |  |  |  |  |
| Intake of protein and kj at 11:30-17:00 |  |  |  |  |
| Intake of protein and kj at 17:00-24:00 |  |  |  |  |
| In total, has patient received nutrition support according to individual nutrition support plan? |  |  |  |  |

| **Mobilisation** |  | **Day 1** | **Day 2** | **Day 3** |
| --- | --- | --- | --- | --- |
| Is patient mobilised to walk or sit out of bed ≥20 minutes |  |  |  |  |
| Is patient in need for mobilisation support? |  |  |  |  |
| Is plan for mobilisation developed? What is the plan? |  |  |  |  |
| The duration of mobilisation (minutes) |  |  |  |  |
| Is patient mobilised according to mobilisation plan? |  |  |  |  |

| **Oxygen therapy** | **Day 1** | **Day 2** | **Day 3** |
| --- | --- | --- | --- |
| Arterial oxygen tension (PaO2) |  |  |  |
| Oxygen saturation (SpO2) |  |  |  |
| Does patient have COPD? |  |  |  |
| Is patient in need for oxygen therapy? |  |  |  |
| Oxygen therapy received in liters |  |  |  |
| Has oxygen therapy initiated and monitored according to SpO_2_ level? |  |  |  |
